# Prevalence of pedophilia in a forensic psychiatric population in Switzerland: a comparative study of contact vs non-contact and pedophilic vs non-pedophilic offenders

**DOI:** 10.3389/fpsyt.2025.1713195

**Published:** 2025-12-29

**Authors:** Clara Anselmetti, Céline Renouard, Mathieu Murdocco, Camille Jantzi

**Affiliations:** 1 Faculty of Medicine, Claude Bernard University Lyon 1, Lyon, France; 2 Department of Psychiatry, Hospices Civils de Lyon (HCL), Lyon, France; 3 Forensic Psychiatry Unit, University Centre of Legal Medicine Lausanne-Geneva, Geneva University Hospitals (HUG), Geneva, Switzerland; 4 University of Geneva, UNIGE, Geneva, Switzerland

**Keywords:** forensic psychiatry, pedophilia, child sexual offenders, child sexual abuse material, criminology

## Abstract

**Introduction:**

Sexual offenses against minors represent a major public health and judicial concern. Pedophilia, defined as a persistent sexual preference for prepubescent children, is frequently assessed in forensic psychiatry, particularly in the context of evaluating criminal responsibility and risk offending, yet its clinical significance and relationship to offending behavior remain debated. This study aimed to determine the prevalence of pedophilia among individuals accused of sexual offenses against minors under 14 of age in Switzerland and to compare offenders with and without this diagnosis.

**Methods:**

A retrospective analysis was conducted on 51 male individuals referred for forensic psychiatric evaluation between 2018 and 2021 in Geneva. Data from forensic reports included sociodemographic characteristics, psychiatric history, ICD-10 diagnoses, and offense details. Comparisons were performed between individuals accused of contact versus non-contact offenses, and secondarily between those with and without pedophilia.

**Results:**

Pedophilia was diagnosed in 58.8% of participants. This diagnosis was associated with higher rates of personality and substance use disorders. However, no significant differences were found between contact and non-contact offenders regarding the prevalence of pedophilia and no distinctive profile emerged within the pedophilic group.

**Discussion:**

These findings confirm the high prevalence of pedophilia in forensic populations and underscore their clinical heterogeneity. They highlight the need for comprehensive, individualized forensic assessments integrating diagnostic, contextual, and dispositional factors, and suggest that future research should further examine the interaction between pedophilic interests, self-control, and situational opportunities.

## 1 Introduction

Sexual offenses against minors represent a major public health and societal concern due to their profound and lasting psychological consequences for victims, including an increased risk of depression, anxiety, post-traumatic stress, and suicidal behaviors (1–5). Understanding the profiles and motivations of perpetrators is therefore crucial to improve prevention and risk assessment strategies.

Among individuals who commit such offenses, a variable proportion meet criteria for pedophilia, defined by the ICD-10 as a persistent sexual preference for prepubescent children (6). It is important to distinguish pedophilia from the sexual offense itself: not all individuals who commit sexual offenses against minors are pedophilic, and conversely, some individuals with pedophilic interests never act on them (7, 8).

Epidemiological studies estimate the prevalence of pedophilia in the general population to be around 3–5%, with broader ranges of 2–24% reported depending on diagnostic criteria (DSM vs ICD), assessment methods (self-report, clinical interview, phallometric testing), and the populations studied (community, student, or offender samples) (9). Among sexual offenders, pedophilia is considerably more prevalent than in the general population (8). Studies using phallometric testing have shown that pedophilia is particularly frequent among extrafamilial offenders with multiple victims (10). Other research indicates that among users of child sexual abuse material (CSAM), about 61% meet criteria for pedophilia compared to 35% among those who commit contact offenses, suggesting a stronger association between pedophilia and non-contact offending (11).

Beyond prevalence, several studies have described the sociodemographic and psychological characteristics of pedophilic individuals. Overall, pedophilic offenders are predominantly male, often older, single, and may present social isolation and employment instability (12, 13). They frequently exhibit psychiatric comorbidities, including mood or anxiety disorders and, in some cases, personality, substance-use disorders or others paraphilia (12, 26).

Neuropsychological and clinical studies further suggest that pedophilia as a sexual preference should be differentiated from the propensity to offend (14). Schiffer et al. (2017) found that pedophilic individuals, whether or not they had committed offenses, shared similar cognitive and affective profiles, whereas offending behavior was more closely related to impulsivity, antisocial traits, or situational factors (15). Consistently, Seto (2009) proposed a motivational model in which pedophilic interests interact with opportunity and self-regulation deficits to determine the risk of sexual offending (8). Moreover, a Swiss study of individuals convicted for CSAM found that these users displayed sociodemographic and criminological profiles distinct from those of contact child molesters, a finding consistent with broader reviews of offender typologies (16, 17). For example, Neutze et al. (2011) showed that CSAM offenders tend to be younger, better educated, and more often employed, while contact offenders are typically older, less employed, and more often known to the justice system, though both groups present elevated psychological distress (18). These profile differences justify the need to compare contact and non-contact offenses.

From a forensic perspective, the diagnosis of pedophilia raises complex questions regarding criminal responsibility. In Swiss jurisprudence, psychiatric disorders such as pedophilia may justify a reduction in criminal responsibility only when they significantly impair cognitive or volitional capacities at the time of the offense, and when the offender’s mental state markedly deviates from both the general population and comparable delinquent groups. However, the mere presence of a diagnosis does not automatically entail diminished responsibility, emphasizing the need for individualized forensic assessments that take into account the degree of impulse control, comorbidity, and situational factors.

The present study provides a descriptive and comparative overview of individuals undergoing forensic psychiatric evaluation in Switzerland for sexual offenses against minors under 14 of age; It focuses on the prevalence of pedophilia and associated psychiatric and sociodemographic characteristics, comparing contact and non-contact offenders, and examines whether a distinctive profile differentiates pedophilic from non-pedophilic individuals. Accurate characterization of offender profiles may help improve risk assessment and identify factors associated with escalation from non-contact to contact offending. We hypothesized that (1) the overall prevalence of pedophilia in this forensic population would be higher than in the general population; (2) non-contact offenders would show a higher prevalence of pedophilia than contact offenders; and (3) certain sociodemographic and psychiatric characteristics would distinguish pedophilic from non-pedophilic offenders.

## 2 Materials and methods

### 2.1 Population

This study is a retrospective analysis based on psychiatric forensic reports conducted between January 1, 2018, and December 31, 2021, at the Forensic Psychiatry Unit (FPU) of the Geneva University Hospitals. The study period was chosen to ensure consistency in the use of the 10th edition of the International Classification of Diseases (ICD-10), prior to the gradual adoption of ICD-11, which came into effect on January 1, 2022 (6, 19, 20). This timeframe also ensured a sufficient number of cases for statistical analysis and allowed categorization of offenses as either contact or non-contact for descriptive analysis.

### 2.2 Sample size calculation

To ensure adequate statistical power to detect a difference in pedophilia prevalence between the two groups (contact and non-contact sexual offenses), we performed a sample size calculation based on prevalence data from previous literature (11, 21). Assuming a 80% statistical power and a 5% significance level, we hypothesized a prevalence of 61% in the non-contact group and 35% in the contact group. The required sample size was calculated using the following formula:


$$n=Z^{2}\times p\left(1–p\right)/d^{2}$$

where *Z* is the z-value corresponding to a 95% confidence interval, *p* is the estimated prevalence for each group (0.61 for the non-contact group and 0.35 for the contact group), and *d* is the minimum detectable difference (0.20 for a 20% difference). Based on this formula, approximately 23 subjects were needed in the non-contact group and 22 in the contact group to detect a significant difference. The corresponding confidence intervals for both groups were also calculated using the formula:


CI = p ± Z × √(p(1–p)/n)

where *p* is the estimated sample prevalence, *Z* is the z-value for a 95% confidence level (1.96), and *n* is the sample size for each group. The resulting confidence intervals were (0.15–0.55) for the contact group and (0.41–0.81) for the non-contact group.

### 2.3 Inclusion criteria

Participants included in this study were male aged 18 or older who underwent a psychiatric forensic evaluation at the FPU for sexual offenses against minors under the age of 14. Inclusion criteria consisted of being accused of a sexual offense involving either contact (such as sexual coercion or rape) or non-contact acts (including voyeurism, exhibitionism, online grooming, or the viewing, distribution, or production of CSAM) committed against a minor under the age of 14. Exclusion criteria included individuals who were themselves minors at the time of the offense, unless they had also committed offenses as adults. Sexual acts involving minors aged 14 and older were excluded, given that the concept of pedophilia is primarily associated with victims under the age of 14. Although female subjects were not explicitly excluded, no psychiatric evaluation files involving women were available during the study period. Thus, out of an initial population of 126 evaluated individuals, a total of 75 were excluded.

### 2.4 Data collected

Forensic psychiatric evaluations were conducted through individual interviews with the examinee (**Table 1**). In some cases, two experts co-authored the report and jointly attended the interviews. The evaluators were psychiatrists trained in forensic psychiatry and practicing within the FPU. The diagnosis of pedophilia was established according to the clinical criteria defined in the International Classification of Diseases, 10th Revision (ICD-10), without the use of standardized assessment tools (e.g., phallometric testing or specific questionnaires) (6). The evaluation was therefore based on the clinical interview, review of judicial records, and available anamnesis.

**Table 1:** General characteristics of individuals accused of sexual offenses against minors.

Characteristics of the accused	N, (%)
Mean age in years	
(± SD; median [IQR])	38.7 (± 14.1; 38.0: IQR 29.0-45.0)
18-30	15 (30.6)
31-50	26 (53.1)
≥ 51	8 (16.3)
Missing data	2 (3.9)
Mean duration of education in years	
(± SD; median [IQR])	13.5 (± 4.2; 14.0: IQR 11.0-16.0)
≤ 10	10.0 (20.0)
11 à 15	26.0 (52.0)
≥ 16	14 (28.0)
Missing data	1 (1.9)
Professional contact with minors under 14	3 (6.0)
Missing data	1 (1.9)
Relationship status	> 6 months:29 (59.2)
single or < 6 months	20 (40.8)
Missing data	2 (3.9)
Has dependent children	27 (54.0)
Missing data	1 (1.9)
Personal history of childhood sexual trauma	12 (24.0)
Missing data	2 (0.02)
Criminal history	26 (51.0)
Missing data	0 (0.0)
Type of criminal history	
Against individuals aged ≥ 14	7 (28.0)
Against minors under 14	6 (24.0)
Nonsexual	10 (40.0)
Offenses related to child sexual abuse material	2 (8.0)
Missing data	1 (0.04)
Psychiatric history	31 (60.8)
Missing data	0 (0.0)
Psychiatric diagnoses	
Personality disorder	10 (19.6)
Psychosexual development disorder	7 (13.7)
Substance use disorder	6 (11.8)
Paraphilic disorders other than pedophilia	8 (15.7)
Pedophilia	30 (58.8)
Intellectual disability	1 (2.0)
Mood disorder	9 (18.4)
Adjustment disorder	2 (0.04)
Mental state alteration	
Missing data	
N	**51 (100.0)**

Bold values indicate the total number of subjects in each group.

#### 2.4.1 Sociodemographic characteristics of the accused

Several variables were collected in this study. The age of the accused at the time of the first alleged offense was categorized into three distinct groups: 18–30, 31–50, and 51 or older, to reflect distinct developmental and socio-professional stages (early adulthood, midlife, and older adulthood) that may influence offending patterns. For individuals who began their alleged offenses as minors but continued them into adulthood, the age of majority was considered, and these individuals were categorized within the 18–30 age group.

Educational level was recorded as the number of schooling completed after the first year of primary school, excluding repeated , and grouped into three categories: 10 or fewer, 11 to 15, and 16 or more. These cut-offs are aligned with commonly used educational stratifications in European population-based studies and reflect meaningful differences in academic achievement and access to social and occupational opportunities. Regarding the occupation of the accused, a distinction was made between those whose profession involved contact with minors under 14 and those without such contact.

Marital status was classified as “in a relationship” if the individual was cohabiting with a partner for at least six months. The presence or absence of biological or adopted children was also recorded.

#### 2.4.2 History of childhood sexual trauma

The presence of childhood sexual trauma, defined as a history of sexual assault or rape reported in the forensic file, was recorded.

#### 2.4.3 Criminal history

Criminal history was documented and categorized by offense type: sexual offenses involving persons aged 14 and over, sexual offenses involving minors under 14, offenses related to CSAM, and non-sexual offenses.

#### 2.4.4 Psychiatric history and diagnoses

The variable psychiatric history referred exclusively to non-sexual disorders diagnosed prior to the alleged offense (mood disorders, post-traumatic stress disorder, psychotic disorders, or substance use disorders) and which were resolved or in remission at the time of the forensic evaluation. In contrast, the variable psychiatric diagnoses included all psychiatric disorders identified during the forensic assessment, whether chronic or newly diagnosed. For analytical purposes, psychiatric diagnoses were classified into the following categories: personality disorders, psychosexual development disorders, substance use disorders, paraphilic disorders (pedophilia and other paraphilias), intellectual disability, mood disorders, and adjustment disorders. Participants could present with more than one diagnosis, and comorbid conditions were recorded; therefore, categories were not mutually exclusive. Diagnostic categories not reported in the table, such as psychotic disorders, were absent in this sample. All diagnoses were coded according to ICD-10 criteria. Personality disorders were most often reported as mixed, schizoid, or other specified personality disorder. In many cases, the diagnosis was noted without detailed subtyping according to ICD-10 clusters. Consequently, personality disorders were analyzed as a single category, representing the presence of enduring personality pathology regardless of subtype. This approach reflects the heterogeneity inherent to retrospective data collection from forensic reports, in which diagnosis formulations may vary in precision. It therefore aims to provide a descriptive overview rather than an analysis of specific personality traits.

#### 2.4.5 Mental state alteration

Mental state alteration at the time of the offense was considered present only when the forensic psychiatric evaluation concluded there was a diminution of criminal responsibility, in accordance with Swiss medico-legal practice and Federal Supreme Court jurisprudence (ATF 116 IV 273) (22).

#### 2.4.6 Criminal acts

Regarding the nature of the criminal acts, individuals were categorized based on whether they were prosecuted for contact offenses, including attempted rape or sexual assault, in accordance with legal definitions, or for non-contact offenses (**Table 2**). The Swiss Penal Code differentiates non-contact sexual offenses (Articles 194 and 197 SCC), including exhibitionism, voyeurism, and use of CSAM, from contact offenses such as sexual touching (Article 198 SCC) and rape (Article 190 SCC). Attempted sexual assault is legally treated the same as completed assault or rape (23). Individuals with a history of contact offenses who were also charged with offenses involving CSAM were classified in the “contact” group. In line with Swiss legal definitions, this classification relied on the presence or absence of direct physical sexual contact with the victim. The non-contact group therefore encompassed heterogeneous behaviors that vary in interpersonal involvement (e.g., online grooming, voyeurism, CSAM-related offenses). This categorization was retained for analytical and forensic relevance, while acknowledging its clinical and criminological heterogeneity as a limitation of the study.

For this variable, terms such as exhibitionism or voyeurism refer to legally defined behaviors rather than paraphilic disorders. The classification therefore reflects judicial categories rather than diagnostic entities.

#### 2.4.7 Sociodemographic characteristics of the alleged victim

The age of the alleged victim was categorized to correspond to major developmental stages of childhood: early childhood (0–5), prepuberty (6–10), and early puberty (11–13 included). In cases involving multiple alleged victims, the age of the youngest victim was considered. If the accused was alleged to have reoffended multiple times against the same victim, the age at the time of the first alleged offense was retained. The gender of the alleged victims was coded as male, female, or both when the accused was prosecuted for offenses involving victims of both sexes. The relationship between the accused and the alleged victim was classified as either intra-familial or extra-familial. Intra-familial relationships included non-biological ties such as those found in blended families (e.g., adopted children or stepchildren).

Finally, missing data were handled using an available case approach. All forensic reports with limited missing values were retained to preserve the representativeness of the study population. Only one case, which presented extensive missing information, was excluded. Statistical analyses were conducted on the available data only (no imputation performed).

### 2.5 Statistical analyses

Statistical analyses were conducted in collaboration with the Methodological Support Unit of the Geneva University Hospitals using STATA 18.0 BE software. Comparisons were first made between individuals prosecuted for contact versus non-contact sexual offenses, and subsequently between those diagnosed with pedophilia and those without such a diagnosis. Non-parametric Mann-Whitney U tests were used for continuous variables, and Chi-squared or Fisher’s exact tests for categorical variables, depending on sample size. Differences were considered statistically significant at *p*< 0.05, and results are presented with 95% confidence intervals.

## 3 Results

### 3.1 General description of the sample

The total sample included 51 participants who underwent forensic psychiatric evaluation for a sexual offense involving a minor under the age of 14. The mean age was 38.7 (± 14.1; median 38.0, IQR 29.0-45.0). Just over half of the participants were in a relationship lasting more than six months, while almost none held professional positions involving regular contact with minors. The mean duration of education was 13.5 (± 4.2; median 14.0, IQR 11.0-16.0), and approximately half reported having dependent children. From a psychiatric perspective, most individuals presented at least one diagnosis. The most frequent disorders were pedophilia (58.8%) and personality disorders (19.6%), mainly of mixed or schizoid types. Less frequent diagnoses included psychosexual development disorders and substance use disorders. Approximately one quarter of the participants reported a history of childhood sexual abuse. All the results are in **Table 1**.

### 3.2 Type of offense

The distribution of offense types is summarized in **Table 2**. Regarding the nature of the offenses, nearly half of the individuals (49.0%) were prosecuted for contact sexual offenses against minors, including sexual touching or coercion. A substantial proportion (33.3%) were involved in offenses related to CSAM, such as viewing, distributing, or producing explicit content involving minors. A smaller group (5.9%) committed both contact offenses and CSAM-related offenses. Lastly, 11.8% were accused of non-contact sexual offenses, such as exhibitionism or inappropriate communication with minors. A diminished mental state at the time of the offense, was found in 18.4% of the cases (**Table 3**).

**Table 2:** Type of offense categories.

Type of offense	N, (%)
Contact sexual offense against a minor	25 (49.0)
Child sexual abuse material	17 (33.3)
Combination of child contact sexual offense and sexual abuse material	3 (5.9)
Non-contact sexual offenses (no included sexual abuse material)	6 (11.8)
Missing data	0 (0.0)
N	**51 (100.0)**

Bold values indicate the total number of subjects in each group.

**Table 3:** General characteristics of offenders with contact and non-contact sexual offenses.

Characteristics	Contact sexual offenses	Non-contact sexual offenses	p-value
Mean age in years			
(±SD; median: [IQR])	40.3 (± 15.5; IQR 38.5: 30.0-52.0)	36.9 (± 12.3; 35.0: IQR 28.0-44.0)	0.567
18 to 30 years	7 (26.9)	8 (34.8)	0.110
31 to 50 years	12 (46.2)	14 (60.9)	
≥ 51 years	7 (26.9)	1 (4.3)	
Missing data	2 (7.1)	0 (0.0)	
Mean duration of education in years			
(±SD; median: [IQR])	11.8 (± 4.2; 12.0: IQR 10.0-15.0)	15.6 (± 3.2; 15.0: IQR 14.0-18.0)	0.002**
≤ 10 years	10 (37.0)	0 (0.0)	0.004**
11 to 15 years	12 (44.4)	14 (60.9)	
≥ 16 years	5 (18.5)	9 (39.1)	
Missing data	1 (3.6)	0 (0.0)	
Professional contact with minors under 14			
>6months	16 (61.5)	13 (56.5)	0.721
Single or ≤ 6 months	10 (38.5)	10 (43.5)	
Missing data	2 (7.1)	0 (0.0)	
Dependent children	16 (59.3)	11 (47.8)	0.419
Missing data	1 (3.6)	1 (4.3)	
Relationships status			
History of childhood sexual trauma	6 (22.2)	6 (26.1)	0.750
Missing data	1 (3.6)	0 (0.0)	
Has dependent children			
Professional contact with minors under 14	1 (3.7)	2 (8.7)	0.439
Missing data	1 (3.6)	0 (0.0)	
Criminal history	16 (57.1)	10 (43.5)	0.331
Missing data	0 (0.0)	0.(0.0)	
Type of criminal history			
Against individuals aged ≥ 14	4 (26.7)	3 (30.0)	0.879
Against minors under 14	3 (20.0)	3 (30.0)	
Nonsexual	7 (46.7)	3 (30.0)	
Offenses related to child sexual abuse material	1 (6.7)	1 (10.0)	
Missing data	1 (6.2)	0 (0.0)	
Psychiatric history	18 (64.3)	13 (56.5)	0.572
Missing data	0 (0.0)	0 (0.0)	
Paraphilia diagnostic			
Pedophilia	16 (57.1)	14 (60.9)	0.077
Other paraphilia	1 (3.6)	5 (21.7)	0.788
Missing data	0 (0.0)	0 (0.0)	
Others psychiatric diagnoses			
Personality disorder	5 (17.8)	5 (21.7)	0.739
Psychosexual development disorder	5 (17.9)	2 (8.7)	0.436
Sexual preference disorder (non-pedophilic)	1 (3.6)	7 (30.4)	0.016*
Substance use disorder	4 (14.3)	2 (8.7)	0.678
Missing data	0 (0.0)	0 (0.0)	
N	**28 (54.9)**	**23 (45.1)**	

*p<0.05.

**p<0.01.

***p<0.001. Bold values indicate the total number of subjects in each group.

### 3.3 Comparison between contact and non-contact offenders

The results are shown in **Table 3**. The comparison between contact and non-contact offenders revealed no significant difference in age (*p* = 0.567, Mann–Whitney U test).

However, the mean duration of education was significantly higher among non-contact offenders (*p* = 0.002). No differences were observed for relationship status (*p* = 0.721, Fisher’s exact test), presence of dependent children (*p* = 0.419), or professional activity involving minors (*p* = 0.439). Similarly, there were no significant differences in criminal history (*p* = 0.331, Chi-square test) or psychiatric history (*p* = 0.572) between the two groups.

### 3.4 Prevalence of pedophilia

The overall prevalence of pedophilia in the studied population was 58.8% (95% CI: 44.2% to 72.4%) (**Table 1**). No significant difference was observed between the contact and non-contact groups for this prevalence (57.1% vs. 60.9%; p = 0.788) (**Table 3**). However, focusing solely on the 17 participants in the non-contact group accused of child pornography offenses (excluding the 6 individuals accused of other types of non-contact sexual offenses), the prevalence of pedophilia reached 82% (14 out of 17). Fisher’s exact test comparing the “contact” group and the “non-contact (child pornography only)” group yielded a p-value of 0.727, indicating no statistically significant difference between the two.

### 3.5 Comparison between pedophile and non-pedophile groups

The results are presented in **Table 4**. When comparing individuals accused of sexual offenses against minors under the age of 14 who were classified as pedophiles versus those who were not, certain differences emerge, although few reach statistical significance.

**Table 4:** General characteristics of pedophilic and non-pedophilic offenders.

Characteristics	Pedophilic	Non-pedophilic	p-value
Mean age in years			
(±SD; median:[IQR])	39.0 (±13.1; 37.0: IQR 29.5-44.5)	38.3 (±15.6; 40.0: IQR 25.0-47.0)	0.959
18 to 30 years	8 (28.6)	7 (33.3)	0.790
31 to 50 years	16 (57.1)	10 (47.6)	
≥ 51 years	4 (14.3)	4 (19.0)	
Missing data	2 (6.7)	0 (0.0)	
Mean duration of education in years			
(±SD; median:[IQR])	14.0 (±3.2; 15.0: IQR 12.0-16.0)	12.9 (±5.3; 13.0: IQR 10.0-17.0)	0.290
≤ 10 years	5 (17.2)	5 (23.8)	0.867
11 to 15 years	16 (55.2)	10 (47.6)	
≥ 16 years	8 (27.6)	6 (28.6)	
Missing data	1 (3.3)	0 (0.0)	
Relationships status			
>6months	20 (71.4)	9 (42.9)	0.044*
Single or ≤ 6 months	8 (28.6)	12 (57.1)	
Missing data	2 (6.7)	0 (0.0)	
Has dependent children			
Dependent children	18 (62.1)	9 (42.9)	0.179
Missing data	1 (3.3)	0 (0.0)	
Personal history of childhood sexual trauma			
History of childhood sexual trauma	8 (27.6)	4 (19.0)	0.485
Missing data	1 (3.3)	0 (0.0)	
Criminal history			
Professional contact with minors under 14	2 (6.9)	1 (4.8)	0.621
Missing data	1 (3.3)	0 (0.0)	
Criminal history	16 (53.3)	10 (47.6)	0.688
Missing data	0 (0.0)	0 (0.0)	
Type of criminal history			
Against individuals aged ≥ 14	3 (18.8)	4 (19.0)	0.573
Against minors under 14	4 (25.0)	5 (23.8)	
Nonsexual	7 (43.8)	6 (28.6)	
Offenses related to child sexual abuse material	2 (12.5)	2 (9.5)	
Missing data	0 (0.0)	0 (0.0)	
Psychiatric history	19 (63.3)	12 (57.1)	0.656
Missing data	0 (0.0)	0 (0.0)	
Others psychiatric diagnoses			
Personality disorder	6 (20.0)	4 (19.0)	1.000
Psychosexual development disorder	2 (6.7)	5 (23.8)	0.092
Sexual preference disorder (non-pedophilic)	2 (6.7)	6 (28.6)	0.052
Substance use disorder	4 (13.3)	2 (9.5)	0.519
Missing data	0 (0.0)	0 (0.0)	
N	**30 (58.8)**	**21 (41.2)**	

*p<0.05.

**p<0.01.

***p<0.001. Bold values indicate the total number of subjects in each group.

Pedophilic offenders were statistically more frequently in a relationship than non-pedophiles (71.4% vs. 42.9%; *p = 0.044*). Mo significant differences were observed regarding age, education level, or psychiatric comorbidities, including personality, psychosexual development, or substance use disorders. Similarly, there were no significant differences in criminal history or type of prior offenses.

### 3.6 Victim characteristics and alleged victim-offender relationship

The mean age of the alleged victims was 8.1 (± 4.2 ; median 8.0, IQR 5.0-12.0). They were most frequently in the 11–14 age range (38.5%), followed by 30.8% each for 0–5 and 6–10. In terms of gender, most victims were girls (73.5%), 6.1% were boys, and in 20.4% of cases, the accused was alleged to have victimized both male and female minors. Regarding the nature of the relationship between the accused and the alleged victim, 60.0% of the cases involved an extra-familial relationship, whereas 40.0% were classified as intra-familial.

The age of the alleged victims also varies, however, for ten individuals accused of child pornography offenses, the age of the alleged victims was not specified in the expert reports. In our study, although the mean and median ages of the victims do not differ significantly between the contact (7.5 ± 4.1 ; median 7.5, IQR 5.0-11.0) and no contact groups (9.1 ±4.2 ; median 11.0, IQR 5.0-12.0) (*p = 0.302*), the distribution across age categories shows a significant difference (*p = 0.042*). This difference is primarily evident in the 11–14 age group, which is significantly more represented in the non-contact group (61.5% vs. 26.9%). Individuals accused of contact sexual offenses against minors appear to target children aged 6 to 10 more frequently (42.3%), and particularly girls (88.5%). The gender distribution of the alleged victims in non-contact offenses is more evenly split between girls and both sexes (56.5% vs. 34.8%, *p = 0.032*). Finally, it appears that contact offenses are significantly more intra-familial (66.7% vs. 8.7%, *p< 0.001*), while non-contact offenses are almost exclusively extra-familial (91.3%).

Finally, when comparing the two groups, victims of pedophilic offenders were slightly younger than those of non-pedophilic offenders (6.8 ± 4.2 ; median 6.0, IQR 3.0-11.0 vs. 9.2 ± 3.9 ; median 10.0, IQR 5.0-12.5), although this difference did not reach statistical significance (*p* = 0.091). In both groups, victims were predominantly female, representing 72.4% of victims in the pedophilic group and 75.0% in the non-pedophilic group. A smaller proportion of cases involved both male and female victims (24.1% vs. 15.0%), and male-only victims remained rare (3-10%). These distributions did not differ significantly between groups (*p* = 0.597). Regarding the victim-offender relationship, the proportion of intra-familial cases was comparable across groups (41.4% among pedophilic vs. 38.1% among non-pedophilic offenders), while extra-familial situations predominated in both categories (58.6% and 61.9%, respectively). No statistically significant difference was observed (*p* = 0.815).

### 3.7 Key significant findings

Several significant differences were observed between individuals accused of contact versus non-contact sexual offenses. The non-contact group had a significantly higher level of formal education (mean of 15.6) compared to the contact group (mean of 11.8; *p* = 0.002). The distribution of the victims’ ages also differed significantly between groups (*p* = 0.042): non-contact offenses more frequently involved minors aged 11 to 14, whereas contact offenses more often targeted children aged 6 to 10. A significant difference was also observed in the gender distribution of the victims, with a predominance of female victims in the contact group and a more balanced distribution in the non-contact group (*p* = 0.032). Furthermore, contact offenses were significantly more likely to occur in intra-familial contexts (66.7%) compared to non-contact offenses, which were predominantly extra-familial (91.3%; *p*< 0.001). Finally, non-pedophilic paraphilic disorders were significantly more common among individuals in the non-contact group (30.4% vs. 3.6%; *p* = 0.016). In addition, pedophiles were significantly more likely to be in a stable relationship compared to non-pedophiles (71.4% vs. 42.9%; p = 0.044).

## 4 Discussion

This study evaluates the prevalence of pedophilia among individuals accused of sexual offenses against minors who underwent forensic psychiatric assessments in Switzerland, comparing two groups: those accused of contact offenses and those accused of non-contact offenses. Our results indicate a prevalence rate of 58.8%, consistent with previous literature (24). The prevalence was similar for contact offenses (57.1%) and non-contact offenses (60.9%). Among individuals accused of offenses involving CSAM, the prevalence of pedophilia reached 82%, which aligns with earlier findings (11, 21). However, no statistically significant difference was found compared to the contact group. According to Seto’s (2018) motivation-facilitation model, sexual offending emerges from the interaction between a deviant sexual interest (motivation) and situational or dispositional factors such as impulsivity, poor self-control, or access to potential victims (facilitation) (25). From this perspective, the distinction between contact and non-contact offending may reflect opportunity and inhibition rather than the presence or absence of a pedophilic drive.

Our study also identified a high prevalence of psychiatric comorbidities, particularly personality disorders (19.6%) and consistent with findings in incarcerated sexual offenders where Cluster B disorders, especially antisocial and borderline traits, are frequent (24, 26). 26, however, found that substance use disorders were more prevalent among sexual offenders in Turkey (27). These discrepancies could be partly explained by the characteristics of our sample, composed exclusively of individuals evaluated in a forensic setting following legal proceedings, as opposed to incarcerated or treatment-seeking offenders (13).

The presence of pedophilia should thus be interpreted within a broader clinical and forensic framework, considering comorbidity, impulsivity, and control capacities. On a sociodemographic level, no significant differences were observed between contact and non-contact offenders, except for the duration of education, which was higher among non-contact offenders. This observation aligns with previous studies showing that CSAM offenders tend to be younger, more educated, and more computer literate, whereas contact offenders are typically older and more often unemployed (13). Such patterns may reflect different offending opportunities rather than distinct psychopathological mechanisms. However, these findings should be interpreted with caution given the heterogeneity of the non-contact group, which included various behaviors ranging in interpersonal involvement and risk level. This diversity may partly explain the observed differences in education and victim age between groups. Nevertheless, similar patterns have been consistently reported in studies focusing on more homogeneous samples of online offenders, suggesting that these distinctions reflect genuine socio-demographic and situational differences rather than methodological artifacts (13, 18, 28).

Regarding the victims, our findings indicate an average age of 8.1 , with a predominance of female victims (73.5%) and a higher proportion of intra-familial cases among contact offenses. These results are consistent with previous data highlighting that intra-familial abuse typically involves younger and predominantly female victims, whereas non-contact offenses are almost exclusively extra-familial (29, 30).

From a forensic standpoint, understanding how psychiatric disorders interact with cognitive and volitional control remains a key issue in the evaluation of sexual offenders. While our study did not directly assess criminal responsibility, the findings highlight the importance of considering both diagnostic and contextual factors when evaluating offenders. The presence of pedophilia alone cannot determine legal responsibility rather, individual assessments should integrate the degree of impulse control, comorbidity, and situational elements that may influence behavior. In this context, it is therefore the severity of the pedophilic disorder, particularly its impact on self-control and behavioral regulation, that may be more relevant from a forensic perspective.

Finally, this study has several limitations, including the retrospective design, the modest sample size, and potential variability in diagnostic formulation across forensic reports. Another limitation concerns the heterogeneity of the non-contact group, which included different behaviors such as the use of CSAM, online grooming, voyeurism, and exhibitionism. Although these acts differ in interpersonal involvement and underlying motivation, they were grouped according to Swiss legal definitions emphasizing the absence of direct physical contact. This categorization ensured forensic consistency but may reduce the clinical interpretability of between-group differences.

Future research should focus on prospective evaluations with standardized tools to better delineate the relationship between pedophilic preference, self-control, and situational risk factors. Moreover, longitudinal studies could clarify the trajectories linking non-contact and contact offending, contributing to more targeted prevention strategies.

## 5 Conclusion

Taken together, these findings provide a more nuanced understanding of the clinical and forensic characteristics of individuals accused of sexual offenses against minors under 14. The high prevalence of pedophilia, frequently associated with other psychiatric comorbidities such as personality or substance use disorders, emphasizes the need to consider these offenders as a heterogeneous group rather than a homogeneous clinical entity.

Differences observed between contact and non-contact offenses, particularly regarding victim age and relationship context, appear to reflect situational and opportunity-related factors more than distinct psychopathological mechanisms. Beyond its descriptive contribution, this study underscores the importance of comprehensive, multidisciplinary assessments in forensic psychiatry. Such evaluations should integrate diagnostic, contextual, and dispositional dimensions to better inform judgments about criminal responsibility and recidivism risk.

Importantly, these findings contribute to refining clinical and forensic practices by highlighting the necessity of individualized assessment rather than categorical assumptions. Future research should expand on these results through larger, prospective studies using standardized instruments, in order to clarify the dynamic interactions between deviant sexual interests, impulse control, and situational triggers, and to inform evidence-based prevention and intervention strategies.

## Data Availability

The raw data supporting the conclusions of this article will be made available by the authors, without undue reservation.
